# Review of Infections in Immunocompromised Travellers: Epidemiology, Infection Prevention and Management

**DOI:** 10.3390/microorganisms14030721

**Published:** 2026-03-23

**Authors:** Anca-Elena Duduveche, Andreea-Lacramioara Mohorea-Neata, Sorina-Nicoleta Badelita

**Affiliations:** 1 Department of Infectious Diseases, University of Medicine and Pharmacy of Craiova, 200349 Craiova, Romania; 2 Department of Hematology, Fundeni Clinical Institute, 022328 Bucharest, Romania; mohorea.neata@gmail.com (A.-L.M.-N.); sorinabadelita@gmail.com (S.-N.B.)

**Keywords:** immunocompromised, infections, vaccinations, prevention, travel

## Abstract

International travel has increased significantly in recent years. Many travellers are immunocompromised, making them more susceptible to infectious diseases due to their weakened immune systems. This narrative review provides an overview of infection epidemiology, prevention strategies, and clinical management for immunocompromised travellers. We demonstrate the role of pre-travel consultation, emphasizing individualized risk assessment informed by destination-specific epidemiology, immune status, and underlying conditions. Key preventive interventions, including vaccination strategies, antimicrobial prophylaxis, and detailed health counseling, in addition to practical measures that can be taken to minimize infection risk during travel, are addressed. Post-travel surveillance and early recognition of travel-related infections are analyzed, highlighting the importance of prompt symptom evaluation. Effective care for immunocompromised travellers requires a multidisciplinary framework that integrates patients, primary care physicians, infectious disease specialists, and travel medicine experts.

## 1. Introduction

The term “immunocompromised” refers to a state involving a quantitative or functional defect in the immune system, either because of disease or as a result of treatment. The immune deficiency may be primary (congenital, such as hereditary immunodeficiencies) or secondary (acquired, for example, via HIV infection, hematological malignancies, solid or hematopoietic organ transplantation, or immunosuppressive treatments, including corticosteroids, chemotherapy, biological therapies, or radiotherapy). The presence of an immune defect (primary or secondary), determined by disease or treatment, increases the risk of infections or infectious complications.

Over recent decades, advances in medical therapies, including immunosuppressive agents and biologics, have improved survival and quality of life for patients with conditions such as HIV/AIDS, patients with malignancy, those receiving immunosuppressive therapies, and patients who have undergone organ or hematopoietic stem cell transplantation, thereby expanding the population of immunocompromised travellers [1,2].

This growth correlates with the global increase in travellers, as evidenced by the projected 1 billion tourist arrivals by 2013 and over 1.4 billion recorded in 2019, before the COVID-19 pandemic [3,4]. The heightened risk of severe infections from various pathogens must be acknowledged. Despite the documented risks, infection prevention and management in immunocompromised travellers remain challenging. The complexity of customizing prophylactic measures, including vaccinations and chemoprophylaxis, for varying immunosuppressive conditions and travel itineraries exacerbates this issue. A knowledge gap persists due to limited information on non-vaccine-preventable infections and drug interactions, impacting optimal vaccination schedules, efficacy, and safety for immunocompromised populations.

Our narrative review aims to analyse and synthesise the current evidence on infection prevention and management strategies for immunocompromised travellers, with a focus on bacterial, viral, and fungal infections. We aim to clarify which prophylactic measures are most effective and to identify persistent knowledge gaps. Ultimately, our goal is to provide practical guidance to clinicians who offer pre-travel counselling and post-travel care. For transparency and reproducibility, we conducted a specific search of PubMed, Embase, Scopus, and Web of Science. We searched for narrative reviews, systematic reviews, and research only in English between 1993 and 2025. Our search included terms related to “immunocompromised traveller”, “immunocompromised patient”, “travel medicine”, “vaccination”, “pre-travel consultation”, and “post-travel consultation”.

## 2. Epidemiology

Travellers with compromised immune systems are more vulnerable to serious consequences from foodborne and waterborne infections (e.g., *Shigella*, *Listeria*, *Salmonella*, *Campylobacter*, and *Cryptosporidium*), which can become chronic or even fatal [3]. They are also more susceptible to geographically focal infections, including visceral leishmaniasis, talaromycosis in Southeast Asia, coccidioidomycosis and histoplasmosis in the Americas, and tuberculosis in endemic regions. Fungal infections are particularly concerning in those with severe cellular immunodeficiency, such as advanced HIV or transplant recipients [5]. Respiratory viral infections, notably SARS-CoV-2, can raise mortality in this population and present a serious risk for severe illness [3].

Diseases that can be prevented by vaccination, such as influenza, hepatitis, and enteric fever, are less common but are associated with more hospitalizations. This shows how important it is to get vaccinated before travelling, even though immune responses may be weaker [4,6]. It appears that the rates of common travel-related infections may not differ substantially between immunocompetent and immunocompromised travellers. However, immunocompromised travellers are more likely to have severe clinical events and complications from infections [5,7]. These observations highlight the importance of personalized pre-travel counselling, comprehensive preventive strategies, and careful post-travel follow-up for unusual or severe presentations.

The most frequent acute infections among short-term (≤2 weeks) travellers are respiratory infections and traveller’s diarrhoea. Travellers who stay for more than a month face cumulative exposure risks, including higher rates of parasite infections, TB exposure, and diseases that can be prevented by vaccination (Table 1). Chronic infections like hepatitis B and C are more common in people who visit endemic areas frequently.

Adventurous travellers who engage in high-risk activities exhibit distinct infection patterns. Those participating in freshwater activities face an increased risk of schistosomiasis and leptospirosis, as well as exposure to waterborne pathogens. Cave exploration and rural trekking increase exposure to histoplasmosis, rabies, and arthropod-borne diseases [8]. Standard tourism to resort areas has lower overall infection rates but poses risks of foodborne and waterborne illnesses due to dietary indiscretion [9].

The prevalence of infections varies considerably across different immunocompromised populations. Recent cohort studies, both prospective and retrospective, show that the risk of significant infections after transplantation is highest during the first year, with rates ranging from 55–62% across large multicenter and single-center cohorts [10,11,12].

Hematopoietic stem cell transplant (HSCT) recipients show even greater vulnerability, with up to 90% developing at least one significant infection during their treatment course [12]. This happens in the first year, especially in the allogeneic setting. For example, a large Swiss cohort found that 88% of adult allogeneic HSCT recipients developed at least one microbiologically documented infection in the first year, with a mean of 3.66 infections per patient-year [13]. Risk is driven by profound neutropenia, mucosal barrier injury, graft-versus-host disease (GvHD), and delayed immune reconstitution. Infections occur in distinct phases: early (bacterial, HSV), intermediate (CMV, fungal), and late (encapsulated bacteria, VZV) [12].

Patients with haematological malignancies receiving chemotherapy have infection rates of 70–80% during periods of neutropenia, particularly with prolonged or profound neutropenia. Risk factors include duration and depth of neutropenia, mucositis, and prior infections. Intensive regimens almost invariably lead to febrile neutropenia, with bloodstream infections occurring frequently [14,15].

**Figure 1:**
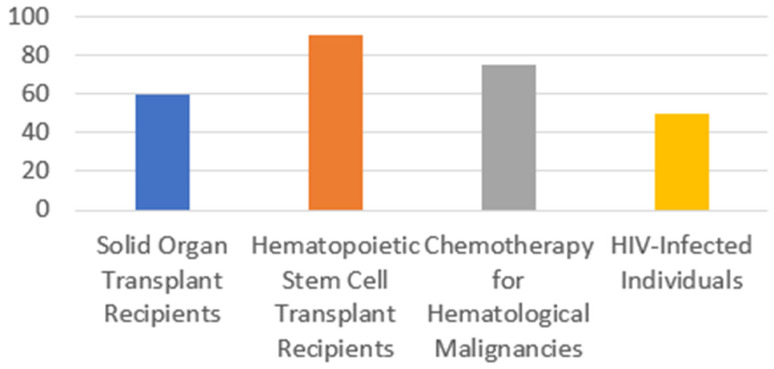
Infection rate in immunocompromised patients.

Individuals with HIV experience infection rates that are inversely proportional to CD4 T-cell counts. The risk of opportunistic infections rises sharply as CD4 counts fall below 200 cells/μL, with specific pathogens (e.g., *Pneumocystis jirovecii*, CMV, *Mycobacterium avium* complex) predominating at lower counts [16]. All of these are summarised in Figure 1 and Table 2.

## 3. Contributing Factors to Increased Risk

### 3.1. Food and Water Contamination

Undercooking food often fails to eliminate vegetative bacteria and parasitic cysts, particularly in regard to meat, eggs, and seafood. As a result, pathogens such as Campylobacter, Salmonella, Shigella, and non-cholera Vibrio species may be transmitted [21]. Cross-contamination between raw and cooked foods, or through contaminated hands and surfaces, constitutes a significant mode of transmission, especially in environments characterized by inadequate hygiene [22]. Improper food storage at ambient temperatures allows rapid bacterial multiplication and toxin production, thereby increasing the risk of foodborne illness [22]. Contaminated water sources, such as municipal supplies that are not adequately treated and ice manufactured from contaminated water, are significant pathways for the spread of infections, including Giardia, Cryptosporidium, Shigella, Salmonella Typhi, and hepatitis A virus [23]. Street-vended foods are often associated with infections due to inadequate refrigeration, poor hygiene, and exposure to environmental pollutants, resulting in an elevated prevalence of multidrug-resistant Enterobacteriaceae [22]. Vibrio species, hepatitis A, and norovirus are all associated with raw or undercooked seafood, particularly in coastal areas [24]. Fresh foods, especially leafy greens and fruits, can become contaminated when they are watered with untreated water or touched by unhygienic hands, which facilitates the spread of enteric bacteria and protozoa [24].

### 3.2. Sexual Risk Behaviors

Travel is linked to increased sexual risk-taking, characterised by more frequent new partnerships and reduced condom use, often shaped by alcohol use, anonymity, and cultural context. Regional differences in sexually transmitted infection (STI) prevalence further influence the risk of contracting STIs; in sub-Saharan Africa, Southeast Asia, and the Western Pacific, for example, there are higher rates of HIV, hepatitis B, syphilis, and antimicrobial-resistant gonorrhea. Certain traveller groups, including men who have sex with men, backpackers, and those visiting friends or relatives, are especially at risk of STI acquisition [25]. The risk is further increased by shorter trip durations, lack of pre-travel consultation, and lack of vaccination [25].

Pre-travel consultation for sexually transmitted diseases (STDs) involves a systematic assessment of individual risk, personalised counselling, and implementation of active and passive preventive measures, according to the recommendations of the Centers for Disease Control and Prevention (CDC). The risk assessment has to include a detailed sexual history, which is obtained using the “Five P’s” approach (partners, practices, protection, history of STDs, pregnancy prevention), with non-judgmental and culturally appropriate language [3]. There is also a need to identify high-risk behaviours (sex with new partners, unprotected sex, alcohol/drug use, use of sex socialising applications).

According to the CDC, active measures such as vaccination against hepatitis A, hepatitis B, and HPV are recommended, but also screening for HIV and treatable STDs (chlamydia, gonorrhea, syphilis, trichomonas) should be performed, with the patient being informed about the tests performed and those available [3]. Into the consultation should be discussed the HIV pre-exposure prophylaxis (PrEP) for individuals at high risk and also prescribed HIV post-exposure prophylaxis (PEP) and emergency contraception, with clear instructions for initiation within <72 h of exposure [3,25].

Passive measures include counselling on correct and consistent condom use, reducing the number of partners, avoiding sex with minors, or engaging in illegal activities [3]. Education of the patient about STD symptoms and the importance of post-travel testing, as well as avoiding exposure to partners upon return, is also very important.

### 3.3. Medical Tourism and Healthcare Exposures

In medical tourism in resource-limited settings, the risk of infection is increased by insufficient sterilization, the reuse of disposable devices, and ineffective infection-control measures. Higher prevalence of surgical site infections and the spread of bloodborne infections like HIV, hepatitis B, and hepatitis C are caused by these factors. Medication and blood-product safety concerns are significant in these settings, with a high prevalence of counterfeit or contaminated medications and inadequately screened blood products. Estimates suggest that a substantial proportion of medications in some regions lack appropriate quality control, and contaminated injectable medications have been linked to outbreaks of bloodborne infections. Blood transfusion risks are increased by insufficient screening for pathogens and a lack of viral inactivation procedures. This makes it even more likely that medical tourists are at risk of being infected with hepatitis B, hepatitis C, and HIV [3].

**Figure 2:**
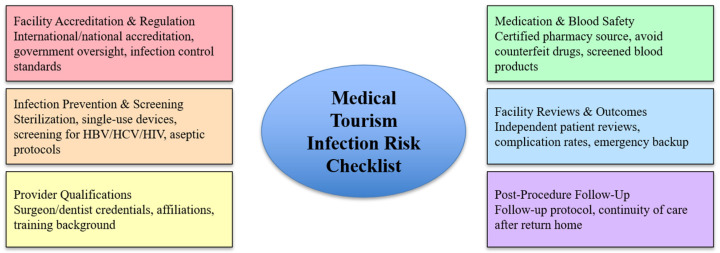
Checklist—Medical Tourism Infection Risk.

The checklist should be systematically reviewed before choosing a destination or provider abroad. Assessing accreditation, evaluating infectious and noninfectious risks, ensuring continuity of care, and fully informing the patient of their rights and associated risks are important (Figure 2).

### 3.4. Recreational Drug Use

High-risk exposure scenarios for bloodborne pathogens are created when travellers who use injection drugs share needles. HIV, hepatitis B, and hepatitis C transmission risks are substantially elevated through contaminated injection equipment [26]. Local drug preparation practices may involve contaminated cutting agents or preparation surfaces, thereby increasing infection risk [27]. Recreational drugs purchased in unfamiliar markets may contain impurities or contaminants that increase susceptibility to infection. Immunosuppressive impurities can weaken host defences, while contaminated diluents may serve as a source of pathogenic organisms [28].

### 3.5. Environmental and Recreational Exposures

Environmental and recreational exposures during travel increase the risk of acquiring waterborne and vector-borne infections not typically encountered in travellers’ home environments. Since both leptospirosis and schistosomiasis are associated with exposure to contaminated water, freshwater activities in endemic areas can increase the risk of these diseases [29]. Vibrio species can infect open wounds exposed to brackish or saltwater, occasionally resulting in severe wound infections. Furthermore, contact with marine life may expose individuals to toxins or cause envenomation by venomous organisms, both of which can occasionally necessitate medical treatment [29]. Contact with wildlife increases the risk of zoonotic disease transmission, while exposure to insects and other arthropods in unfamiliar ecosystems raises the risk of vector-borne diseases, often beyond the protection given by standard preventive measures [3]. A detailed travel and exposure history is essential for risk assessment and diagnosis in returning travellers, as the spectrum of possible infections is highly dependent on specific activities and geographic regions visited (Table 3).

### 3.6. Accommodation and Transportation Factors

Shared accommodation facilities, public transportation, and group tourism activities create opportunities for the transmission of respiratory pathogens. Outbreaks of respiratory infections, including influenza, SARS-CoV-2, and Legionella, have been reported in settings such as hotels, cruise ships, and tour groups, with higher risk among children, older adults, and individuals with comorbid pulmonary conditions [3]. Bed bug infestations in accommodation facilities cause nuisance bites and can lead to secondary bacterial infections through scratching and poor hygiene. Bed bug bites typically present as pruritic, erythematous papules, often in linear or grouped patterns, and scratching can disrupt the skin barrier, predisposing to secondary infections such as impetigo, cellulitis, or lymphangitis [30].

## 4. Pre-Travel Preparation and Risk Assessment

Pre-travel preparation and risk assessment for immunocompromised travellers must be customised and comprehensive, taking into account the traveller’s underlying medical condition, level of immunosuppression, destination-specific risks, and planned activities. The Centers for Disease Control and Prevention (CDC) advises that a detailed pre-travel consultation should include an assessment of disease stability, a review of current medications, and an evaluation of contraindications or interactions with travel-related prophylaxis and vaccines. Collaboration with the patient’s primary or specialty care provider is advised to ensure fitness for travel and to optimize management of the underlying condition during travel [3,5].

### 4.1. Individual Risk Profiling

Developing a detailed itinerary that includes all planned locations and specific regions to be visited, distinguishing between urban and rural areas, the order of destinations, the season, timing, and total travel duration should be the first step in a thorough risk assessment for pre-travel preparation. The reason for travel (e.g., business, tourism, visiting friends and relatives, and humanitarian work) and travel type (e.g., independent, group, luxury, budget, and local accommodations) are important, as these factors influence exposure risk and access to healthcare. Planned activities must be reviewed in detail, with attention to high-risk exposures such as adventure sports, animal contact, freshwater activities, or high-altitude travel, as these may require specific precautions (e.g., rabies vaccination, altitude illness prophylaxis) [3]. Underlying medical conditions should be systematically evaluated, including chronic diseases (e.g., cardiovascular, pulmonary, and diabetes), as these may affect fitness for travel and require tailored management plans. Immunisation history must be reviewed to ensure all routine and travel-specific vaccines are up to date, considering both destination risks and the traveller’s health status. Current medications should be provided, with plans for adequate supply, and potential interactions with travel-related prophylaxis or treatments assessed [31].

### 4.2. Destination-Specific Risk Analysis

Destination-specific risk analysis for immunocompromised travellers is a structured assessment that quantifies the infectious and non-infectious risks at the travel destination, taking into account the traveller’s degree and type of immunosuppression, planned activities, and local epidemiology. This process aims to identify geographically focal infections (e.g., visceral leishmaniasis in certain regions) that can increase the risk of severe outcomes among immunocompromised individuals, and to determine whether specific interventions (e.g., chemoprophylaxis, vaccination, or itinerary modification) are warranted [8].

Chemoprophylaxis selection should be tailored to the destination’s malaria species, resistance patterns, and the traveller’s comorbidities and medications. For regions with chloroquine-resistant *Plasmodium falciparum*, the CDC recommend atovaquone–proguanil (250 mg/100 mg once daily, starting 1–2 days before travel, continued daily during travel, and for 7 days after leaving the area), or doxycycline (100 mg once daily, same schedule, but continued for 30 days after exposure), or mefloquine (228 mg base once weekly, starting 1–2 weeks before travel, continued weekly during travel, and for 4 weeks after exposure) [3].

### 4.3. Vaccinations of Immunocompromised Travellers

Immunocompromised travellers are advised to follow vaccination protocols that maximize protection while lowering the risk of live vaccinations. Immunocompromised travellers should receive all recommended non-live (inactivated or recombinant) vaccines in accordance with routine and travel-specific schedules, as recommended by the Centers for Disease Control and Prevention (CDC) and the Infectious Diseases Society of America (IDSA). This is because these vaccines are generally safe and effective, though immunogenicity may be decreased. Inactivated influenza, hepatitis A and B, pneumococcal, meningococcal, polio (IPV), rabies, and recombinant zoster vaccines are a few examples [32]. Live vaccines (e.g., yellow fever, MMR, varicella) are generally contraindicated in those with severe immunosuppression, but may be considered in select cases (e.g., HIV-infected individuals with CD4 counts ≥200 cells/μL) after careful risk assessment. The IDSA and CDC recommend that, if live vaccines are required for travel (e.g., yellow fever), they should be administered only if the degree of immunosuppression is mild and the risk of disease is high; otherwise, a medical waiver should be obtained [33].

### 4.4. Travel-Specific Vaccinations

#### 4.4.1. Hepatitis A Vaccination

Immunocompromised travellers to areas of high or intermediate hepatitis A endemicity should receive a single dose of hepatitis A vaccine as soon as travel is planned and complete the 2-dose series according to the routine schedule [34,35]. Post-vaccination antibody confirmation (HAV IgG ≥10 mIU/mL) is recommended for immunocompromised individuals whose clinical management depends on knowing their immune status, and for those with HIV infection, testing should occur ≥ 1 month after completing the vaccine series [3].

#### 4.4.2. Hepatitis B Vaccination

For immunocompromised travellers, hepatitis B vaccination requires modified strategies to optimize immunogenicity and protection. Double-dose vaccines are preferred for individuals with HIV and other immunocompromised conditions [35]. Accelerated schedules are appropriate for urgent travel but may yield lower peak antibody levels; completion of the full series is necessary for optimal long-term protection [3].

#### 4.4.3. Typhoid Vaccination

The Centers for Disease Control and Prevention (CDC) and the Advisory Committee on Immunization Practices (ACIP) specifically recommend the injectable Vi capsular polysaccharide vaccine (ViCPS, Typhim Vi) for immunocompromised patients and advise against the use of the live oral vaccine in these individuals [3]. The Infectious Diseases Society of America also recommends the avoidance of live-attenuated oral typhoid vaccine in people with HIV [35]. Inactivated typhoid vaccines provide 50–80% protection in healthy individuals, but efficacy may be reduced in immunocompromised travellers [35].

#### 4.4.4. Meningococcal Vaccination

Immunogenicity of meningococcal vaccines may be reduced in certain immunocompromised populations, particularly those with complement deficiencies or receiving complement inhibitors such as eculizumab or ravulizumab. Antibody responses may be suboptimal in these patients, necessitating consideration of additional protective measures such as antibiotic prophylaxis during periods of high exposure risk [36]. For travellers with functional or anatomic asplenia, persistent complement component deficiency, or those on complement inhibitors, a two-dose primary series of MenACWY (doses administered 8–12 weeks apart) is recommended, with subsequent booster doses every 5 years for patients with ongoing risk [36].

#### 4.4.5. Polio Vaccination

Polio vaccination for immunocompromised travellers should always utilize the inactivated polio vaccine (IPV), as the oral polio vaccine is contraindicated due to the risk of vaccine-associated paralytic polio. A single lifetime IPV booster is considered sufficient for adults with prior vaccination, but long-term travellers may be subject to country-specific requirements for documentation and additional boosters [3].

#### 4.4.6. Yellow Fever Vaccination

The yellow fever vaccine is a live attenuated vaccine and is contraindicated in individuals with severe immunosuppression (e.g., organ transplant recipients, those on high-dose immunosuppressive or immunomodulatory therapies, or with thymus disorders associated with abnormal immune function), due to the risk of life-threatening adverse events such as yellow fever vaccine–associated viscerotropic disease (YEL-AVD) and neurologic disease (YEL-AND) [3]. The United States Centers for Disease Control and Prevention (CDC) and its Advisory Committee on Immunization Practices (ACIP) recommend that such travellers be strongly discouraged from visiting yellow fever–endemic areas. If travel is unavoidable, a medical waiver should be provided, and the traveller must be counselled on the risks of being unvaccinated and the possibility of entry denial or quarantine [3,37]. No inactivated yellow fever vaccine is currently available for individuals with contraindications. Family members of immunocompromised individuals who are not immunocompromised may safely receive the vaccine.

#### 4.4.7. Rabies Vaccination

Immunocompromised travellers should receive a 3-dose series of rabies vaccine administered intramuscularly on days 0, 7, and 21 or 28, rather than the 2-dose series used for immunocompetent persons. This adjustment is based on evidence that immunocompromised individuals may have a reduced and less durable antibody response to rabies vaccination [3].

#### 4.4.8. Influenza Vaccination

Immunocompromised travellers should avoid travel during periods of high influenza activity at their destination, if possible. Influenza seasonality varies by hemisphere and is less predictable in the tropics, so risk assessment should be individualized. If travel is unavoidable, vaccination should be administered at least two weeks before departure. All immunocompromised individuals receive an age-appropriate inactivated influenza vaccine (IIV) or recombinant influenza vaccine (RIV), but not live attenuated influenza vaccine (LAIV), due to the risk of vaccine-associated disease. Timing of vaccination should be optimized, ideally before initiation of immunosuppression or during periods of lowest immunosuppressive therapy, when feasible. For solid-organ transplant recipients, high-dose or adjuvanted IIV may improve immunogenicity; however, there is no clear preference over standard-dose IIV or RIV for most immunocompromised adults [38].

#### 4.4.9. Pneumococcal Vaccination

A single dose of PCV20, or PCV15, followed by PPSV23, is recommended regardless of travel destination if they have not previously received a pneumococcal conjugate vaccine or if their vaccination history is unknown.

#### 4.4.10. Recombinant Zoster Vaccination 

Is recommended for immunocompromised travellers aged ≥ 19 years to prevent herpes zoster and its complications, regardless of travel plans [3].

### 4.5. Accelerated Vaccination Schedule

Accelerated vaccination schedules are a validated strategy for immunocompromised travellers who require rapid protection before imminent travel.

Partial vaccination prior to travel confers meaningful protection, and travellers should be counselled to complete the series upon return for durable immunity. Emergency vaccination protocols prioritise vaccines based on destination-specific risks and individual susceptibility, with hepatitis A and B being high priority for travellers to endemic regions. For immunocompromised travellers, post-vaccination serologic testing is recommended to confirm seroprotection, and immune globulin may be co-administered with hepatitis A vaccine if travel is imminent and immune response is uncertain [3,35,39,40]. We present tables comparing classic and accelerated vaccination schedules for immunocompetent and immunocompromised travellers (Table 4 and Table 5).

### 4.6. Travel Logistics and Preparedness

**Figure 3:**
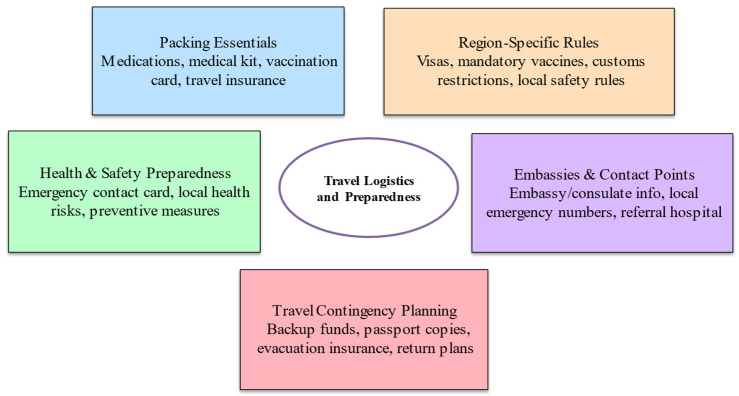
The travel logistics & preparedness flowchart can serve as both a clinical tool (for pre-travel counselling) and a patient-facing checklist.

A structured checklist approach ensures that essential items, regulatory requirements, and contingency plans are addressed systematically during pre-travel counselling. Figure 3 summarises the key domains of travel logistics and preparedness in a flowchart format, providing a practical tool for clinicians and a user-friendly reference for patients.

Immunocompromised travellers should bring an extra supply of all essential medications, ideally enough for the entire trip, plus additional quantities to cover unexpected delays. Medications should be kept in their original packaging with clear labels to facilitate identification and avoid issues at customs or border crossings [3,41].

Medications requiring refrigeration (e.g., certain biologics) should be transported in insulated containers with cold packs. Travellers should verify storage requirements for each drug and plan for contingencies if refrigeration is unavailable. All medications should be stored in carry-on luggage to prevent loss or damage [42].

Travellers should carry a comprehensive medication list, including generic and brand names, dosages, and indications. A physician’s letter detailing the immunosuppressive regimen, underlying condition, and necessity of the medications is recommended. The letter should explicitly state the need for carrying a buffer supply (30–50% extra) due to the risk of travel delays. This documentation can be critical for customs and border officials, and in medical emergencies [42]. Countries in the Middle East (such as the United Arab Emirates, Saudi Arabia, and Qatar), Southeast Asia (including Singapore and Thailand), and some in Africa and South America are known to have stringent controls on the importation of prescription drugs, especially those considered controlled substances or those with immunosuppressive or psychotropic properties. Requirements may include advance notification, import permits, and detailed documentation [43].

Personal protection kits for immunocompromised travellers should include antibacterial hand wipes or alcohol-based hand sanitiser (≥60% alcohol), face masks, insect repellents (for skin and clothing), sun protection (sunscreen, protective clothing, sunglasses) and water purification methods. It can include a thermometer, pain relievers, and basic medications for managing minor illnesses that might otherwise require healthcare facility visits in questionable environments.

Telemedicine access should be planned by identifying internationally available platforms and ensuring that the traveller has the necessary devices and connectivity. The CDC Yellow Book recommends preloading relevant apps and confirming that the traveller’s home providers offer remote consultation options [3].

Immunocompromised travellers require comprehensive coverage for emergency medical care, medical evacuation, access to specialised medical centers, telemedicine services, and buffer supplies of immunosuppressive medications. Insurance policies should be scrutinized for exclusions related to pre-existing conditions, as many claims are denied for this reason. Direct discussion with the insurer prior to travel is recommended to clarify coverage for immunosuppressive therapy and related complications [3].

## 5. During Travel

### 5.1. Hygiene Practices

Recommended hygiene practices for immunocompromised travellers include frequent, thorough handwashing with soap and water, especially after contact with public surfaces or animals and before eating or preparing food. When soap and water are unavailable, it is recommended to use an alcohol-based hand sanitiser containing at least 60% alcohol. Avoid touching the face, particularly in high-risk environments such as airports and public transportation is also important. Mask use in crowded or enclosed spaces is recommended to reduce the risk of respiratory infections.

### 5.2. Food and Water Safety

Food and water safety measures require strict avoidance of raw or undercooked meats, seafood, unpasteurized dairy, and foods from street vendors. It is recommended that only steaming-hot foods, fruits peeled by the traveller, and bottled or canned beverages be consumed. Tap water, ice made from tap water, and untreated water should be avoided. Boiling water for at least one minute is the most reliable method for disinfection; chemical treatments (iodine/chlorine) are less effective against Cryptosporidium [44].

### 5.3. Environmental Awareness

Include avoiding swimming in water that may be contaminated with sewage or animal waste, not swallowing water during recreational activities, and avoiding direct exposure to salt water for those with liver disease due to Vibrio risk. Sun protection is essential due to the increased risk of skin cancer and photosensitivity associated with certain medications. The use of broad-spectrum sunscreen and protective clothing is important [44].

### 5.4. Managing and Recognising Warning Signs of Illness

Involve monitoring for fever, diarrhoea, respiratory symptoms, or other acute changes (Table 6). Immunocompromised travellers should be educated to promptly report any illness and provide travel history to healthcare providers, as infections may present atypically and can be severe or chronic. Because infections may present atypically, clinicians should be aware that immunocompromised patients may not mount a classic inflammatory response. For example, neutropenic or immunosuppressed individuals may have minimal fever or localizing signs despite significant infection [45].

### 5.5. Seeking Medical Care

Include identifying clinics or hospitals at the destination that can manage immunocompromised patients, knowing how to access embassy resources, and purchasing supplemental insurance for medical care and evacuation. Early medical evaluation is critical for any illness during or after travel.

A rapid post-exposure management for rabies in immunocompromised individuals is needed and consists of immediate and thorough washing of the wound with soap and water, followed by application of a virucidal agent, but also administration of rabies immunoglobulin (RIG) 20 IU/kg and administration of five doses of rabies vaccine on days 0, 3, 7, 14, and 28 [3]. Monitoring of serologic response to observe neutralising antibody titers should be checked 1–2 weeks after completion of the series; if the titer is <0.5 IU/mL, an additional dose should be administered and the test repeated.

## 6. Post-Travel Follow-Up

Post-travel follow-up care involves a structured evaluation to identify and manage travel-associated illnesses, particularly in those returning from low- and middle-income countries or with high-risk exposures. The process begins with a detailed, itinerary-specific history, including destinations, duration, activities, exposures (e.g., food, water, animals, insect bites) [4].

A focused physical examination should be performed, guided by symptoms and exposure history. Immunocompromised patients may present atypically or with subtle findings. Tailored testing should include early and repeated malaria diagnostics, blood cultures, and targeted serologies/PCR for regionally relevant pathogens. Screening for tuberculosis (using IGRA or chest radiograph, as skin testing may be less sensitive) is particularly important after traveling to endemic areas. Consideration of multidrug-resistant organisms is heightened, and stool ova and parasite test, cultures, and serologies for strongyloidiasis and schistosomiasis are prioritised in those with relevant exposures [4,8,46].

Infection control precautions are necessary for suspected high-consequence or transmissible infections (e.g., viral hemorrhagic fevers, SARS-CoV-2). Consultation with infectious disease or tropical medicine specialists is recommended for severe, complicated, or unclear cases. Post-travel visits also provide an opportunity for preventive counselling regarding future trips and for addressing ongoing or new symptoms that may arise after return, as delayed presentations are not uncommon.

Individuals requiring post-travel screening even if asymptomatic include:

- Health care workers or those with prolonged residence (>6 months) in high TB prevalence areas (TB screening 8–10 weeks post-return) [3]
- Travellers with freshwater exposure in schistosomiasis-endemic regions (schistosoma serology) [3]
- Those with frequent barefoot exposure to contaminated soil (strongyloides serology) [3]
- Travellers with sexual, medical, or injection drug exposures (STI/bloodborne pathogen screening) [3]
- Long-term travellers, expatriates, humanitarian workers, and those visiting friends/relatives in endemic areas (CBC, stool ova and parasite, serology for region-specific pathogens) [3,47]

Routine screening of short-term asymptomatic travellers is generally not recommended unless specific risk factors are present, due to low yield and risk of false positives [3]. The Infectious Diseases Society of America recommends stool PCR and culture for travellers with fever or bloody diarrhoea, and targeted serology based on exposure history. Tuberculosis screening should be performed 8–10 weeks after return for those with high-risk exposures, using IGRA or TST [3,47]. A structured, risk-based approach ensures prompt identification and management of serious travel-related infections, while minimising unnecessary testing in low-risk individuals [48].

## 7. Future Directions and Research Gaps in Travel Medicine for Immunocompromised Travellers

### 7.1. Evidence-Based Vaccination Strategies

Current recommendations for immunocompromised travellers are largely extrapolated from expert opinion and limited data, with a lack of robust, prospective studies on vaccine immunogenicity, safety, and efficacy in this population. Research on the best times, dosages, and schedules for both live and inactivated vaccines is required, as is the creation of substitute plans for people who cannot receive live vaccinations [49].

### 7.2. Pandemic Preparedness

The COVID-19 pandemic revealed many deficiencies in travel medicine protocols for immunocompromised populations, notably reduced vaccine efficacy and increased illness severity. Future pandemic preparedness must incorporate personalised protocols for immunocompromised travellers. These should include:

- up-to-date vaccination with additional booster doses, recognising that immune responses may be suboptimal and vary by underlying condition and vaccine type;
- considering pre-exposure prophylaxis with monoclonal antibodies and early access to oral antivirals for those with poor vaccine response;
- maintaining strict non-pharmaceutical interventions such as masking, avoiding poorly ventilated spaces, and advising close contacts to adhere to similar precautions;
- developing emergency plans for medical evacuation and care abroad, including insurance coverage and access to specialised care; and
- including immunocompromised populations in future vaccine and therapeutic trials to generate robust efficacy and safety data [50,51,52,53].

### 7.3. Access to Care and Emergency Planning

Research is necessary to identify optimal strategies for emergency planning, international access to specialised care, and the effects of telemedicine and digital health tools on this demographic [54]. The CDC maintains the necessity of identifying clinics or hospitals abroad that can care for immunocompromised patients, purchasing supplemental insurance for medical evacuation, and knowing how to access embassy resources [3].

### 7.4. Impact of New Therapies on Travel Advice

As more people use biological therapies such as monoclonal antibodies, checkpoint inhibitors, and targeted small molecules, travel medicine faces new challenges. These agents have different ways of working and different risks of infection, which may mean that travel advice needs to be tailored to each person. Chimeric antigen receptor T-cell (CAR-T) therapy and other cellular immunotherapies induce prolonged periods of significant immunosuppression, characterised by distinct patterns of infection susceptibility. Patients undergoing these therapies necessitate distinct travel guidelines that may markedly diverge from conventional immunosuppression management [32,55].

### 7.5. Next Generation Vector Control

Wearable spatial repellents, clothing treated with insecticides that last longer, and smart traps linked to exposure dashboards are new environmental and vector control innovations important for travel medicine in people with weak immune systems. Field trials of volatile pyrethroid spatial repellents (VPSRs) and etofenprox-treated clothing have shown a big drop in Anopheles mosquito landings (protective efficacy 61–95%). The best protection comes from using both at the same time, but the effectiveness may go down over time and with adherence problems [56].

### 7.6. Implementation of Science and Equity

This includes the need for pragmatic trial designs that compare standard versus algorithm-guided pre- and post-travel care. Such trials should incorporate point-of-care serology and artificial intelligence-based risk scores to tailor interventions by immune status and destination, as the complexity of pretravel assessment and the heterogeneity of immunosuppression require individualised, data-driven approaches [42]. Algorithm-guided care can standardise risk stratification, optimize vaccine and prophylaxis selection, and facilitate timely post-travel follow-up, addressing the current lack of uniform guidelines and variability in practice [57].

Equity solutions must close the gaps in access for travellers from low- and middle-income countries, migrants, and humanitarian workers who are on immunosuppressive drugs. This includes making and using digital tools for risk assessment, education, and care navigation that work in any language, as well as adding travel health to regular primary and specialty care for immunocompromised populations [53,58].

### 7.7. Artificial Intelligence and Personalization

Multimodal risk engines leveraging artificial intelligence (AI) are increasingly capable of integrating diverse data types—such as medication history, laboratory results, genotype, microbiome, travel itinerary, and seasonality—to generate individualized risk predictions for conditions like venous thromboembolism (VTE), malaria, severe dengue, traveller’s diarrhea, and vaccine failure. For instance, combining clinical and genetic risk factors has been shown to greatly improve the prediction of VTE risk. Polygenic risk scores and clinical models work better together than either one alone [59]. In travel medicine, machine learning models that use real-world data like demographics, destination, and vaccination history can make malaria risk assessments more personal and improve chemoprophylaxis recommendations, which cuts down on unnecessary prescriptions [60].

Digital twin technology is becoming a way to test out prophylaxis, vaccines, and drug interactions before traveling. Digital twins integrate multi-omics, behavioural, and environmental data to create dynamic, patient-specific simulations, enabling predictive analytics for disease risk, treatment response, and drug-drug interaction modelling [60]. These virtual representations can be used to test different preventive strategies and optimize interventions before actual exposure, supporting precision travel medicine.

## 8. Conclusions

Immunocompromised travellers face a disproportionate burden of infection, driven by destination epidemiology, the intensity and type of immunosuppression, and exposure behaviours. Our review shows that the risk is not uniform: hematologic malignancies, B-cell-depleting therapies, and transplant recipients entail distinct vulnerabilities. Outcomes improve when prevention and management are individualized. With individualized planning, interaction-savvy prophylaxis, and rapid, protocolized responses to illness, most immunocompromised patients can travel safely. The field now needs implementation studies and equitable tooling to make this standard—not the exception—across diverse settings.

## Figures and Tables

**Table 1:** Main differences in infectious risks between short-term (≤2 weeks) and long-term (>1 month) immunocompromised travellers.

Feature	Short-Term Travellers (≤2 Weeks)	Long-Term Travellers (>1 Month)
1. Predominant infections	Acute: traveller’s diarrhoea, respiratory tract infections	Cumulative/chronic: parasitic infections, tuberculosis, viral hepatitis
2. Exposure profile	Limited exposure, mainly food- and water-borne	Prolonged/repeated exposure, close contact with the local environment
3. Vaccine-preventable diseases	Less frequent (e.g., typhoid fever, influenza)	More frequent (e.g., hepatitis A/B, yellow fever, measles)
4. Opportunistic/fungal pathogens	Very low incidence	Increased risk (e.g., histoplasmosis, other endemic mycoses)
5. Clinical implications	Usually self-limiting but can be severe in immunocompromised hosts	Higher risk of severe disease, latent infections, and long-term complications

**Table 2:** The epidemiology of infections in immunocompromised patients.

Patient Group	Common Pathogens	Key Risk Factors
Solid Organ Transplant Recipients	*Escherichia coli*, *Klebsiella* spp., *Pseudomonas aeruginosa*, *Staphylococcus aureus*, *Enterococcus* spp., CMV, HSV, VZV, EBV, *Candida* spp., *Aspergillus* spp.	Intense immunosuppression, surgical factors, and comorbidities [10,17]
Hematopoietic Stem Cell Transplant Recipients	*Streptococcus pneumoniae*, *Staphylococcus aureus*, *Pseudomonas aeruginosa*, *Candida* spp., *Aspergillus* spp., CMV, HSV, VZV, respiratory viruses	Neutropenia, mucosal injury, GvHD, delayed immune reconstitution [17,18]
Chemotherapy for Haematological Malignancies	*Staphylococcus aureus*, *Streptococcus* spp., *Escherichia coli*, *Pseudomonas aeruginosa*, *Klebsiella* spp., *Candida* spp., *Aspergillus* spp., HSV, CMV, respiratory viruses	Depth/duration of neutropenia, mucositis, prior infections [19]
HIV-Infected Individuals	*Streptococcus pneumoniae*, *Mycobacterium tuberculosis*, *Pneumocystis jirovecii*, *Mycobacterium avium complex*, *Cryptococcus neoformans*, *Toxoplasma gondii*, CMV, HSV, VZV	CD4 count, ART status, comorbidities [20]

**Table 3:** Activity-pathogen correlations. Recreational and environmental activities during travel significantly influence the risk of exposure to waterborne and vector-borne pathogens. A correlation matrix highlights the relationships between activity types and associated pathogens, which are useful for risk assessment and pre-travel counselling.

Activity/Exposure	Main Pathogens	Type of Infection	High-Risk Regions	Relevant Clinical Observations
Swimming in freshwater	*Schistosoma* spp., *Leptospira* spp.	Waterborne	Africa, Asia, South America	Schistosomiasis, leptospirosis
Swimming in seawater	*Vibrio* spp., norovirus	Waterborne	Tropics, coastal zones	Gastroenteritis, skin infections
Consumption of contaminated food/water	*Enterotoxigenic E. coli*, *Salmonella*, *Giardia*, *Entamoeba*, hepatitis A/E	Waterborne	Global, Asia, Africa, Central America	Acute diarrhoea, viral hepatitis
Camping, trekking, safari	*Plasmodium* spp., dengue virus, chikungunya virus, Zika virus, *Rickettsia* spp., Tick-borne encephalitis virus	Vector-borne	Africa, Asia, South America, Eastern Europe	Fever, rash, encephalitis, malaria
Rural/agricultural excursions	*Plasmodium* spp., *Leptospira*, *Brucella*, *Rickettsia* spp., Tick-borne encephalitis virus	Vector-borne/Zoonotic	Africa, Asia, Eastern Europe	Malaria, leptospirosis, brucellosis, and tick-borne encephalitis
Visits to farms/animal contact	*Brucella*, *Leptospira*, rabies virus	Zoonotic	Global	Brucellosis, leptospirosis, rabies
Urban travel	Dengue virus, chikungunya virus, Zika virus, influenza virus	Vector-borne/Respiratory	Asia, South America, Africa	Fever, rash, respiratory infections
Forest hiking	Tick-borne encephalitis virus, *Borrelia*, *Rickettsia*	Vector-borne	Eastern Europe, USA, Russia	Encephalitis, Lyme disease, rickettsioses
Nighttime outdoor activities in the tropics	*Plasmodium* spp., arboviruses	Vector-borne	Africa, Asia, South America	Malaria, viral fevers

**Table 4:** Classic versus accelerated vaccination schedule, for immunocompetent individuals.

Vaccine	Classic Schedule for Immunocompetent Individuals	Accelerated Schedule for Immunocompetent Individuals	Ready for Flight/Vacation Criteria
Hepatitis A	2 doses (0, 6–12 mo)	2 doses (0, 2–4 wks)	2nd dose ≥ 2 wks before travel, no major adverse events
Hepatitis B	3 doses (0, 1, 6 mo)	4 doses (0, 1, 2, 12 mo)	Last dose ≥ 2 wks before travel, anti-HBs ≥ 10 mIU/mL
Yellow Fever	1 dose ≥ 10 days before travel	Not accelerated	Dose ≥ 10 days before travel, no contraindications
Typhoid (inactivated)	1 dose ≥ 2 wks before travel	Not accelerated	Dose ≥ 2 wks before travel, no major adverse events
Meningococcal	1 dose ≥ 10 days before travel	Not accelerated	Dose ≥ 10 days before travel, no major adverse events
Japanese Encephalitis	2 doses (0, 28 days)	2 doses (0, 7 days, ≥1 wk before travel)	2nd dose ≥ 1 wk before travel, no major adverse events
Rabies	3 doses (0, 7, 21/28 days)	3 doses (0, 3, 7 days, ≥1 wk before travel)	3rd dose ≥ 1 wk before travel, no major adverse events
MMR/Varicella	2 doses (0, 28 days)	2 doses (0, 14 days, ≥2 wks before travel)	2nd dose ≥ 2 wks before travel, no major adverse events
Zoster (RZV)	2 doses (0, 2–6 mo)	Not accelerated	2nd dose ≥ 2 wks before travel, no major adverse events

**Table 5:** Classic versus accelerated vaccination schedule, for immunodeficient patients.

Vaccine	Classic Schedule (Immunodeficient)	Accelerated Schedule (Immunodeficient)	Ready for Flight/Vacation Criteria
Hepatitis A	2 doses (0, 6–12 mo) + serology	2 doses (0, 2–4 wks) + serology	HAV IgG ≥ 10 mIU/mL, 2nd dose ≥ 2 wks before travel
Hepatitis B	4 doses (0, 1, 2, 6 mo, 40 μg) + serology	4 doses (0, 1, 2, 6 mo, 40 μg) + serology	Anti-HBs ≥ 10 mIU/mL, last dose ≥ 2 wks before travel
Yellow Fever	Contraindicated (severe)	Contraindicated (severe)	Specialist clearance if considered, neutralizing titer if indicated
Typhoid (inactivated)	1 dose ≥ 2 wks before travel	Not accelerated	Dose ≥ 2 wks before travel, counsel on reduced efficacy
Meningococcal	1 dose ≥10 days before travel	Not accelerated	Dose ≥ 10 days before travel, counsel on reduced efficacy
Japanese Encephalitis	2 doses (0, 28 days) (reduced efficacy)	2 doses (0, 7 days, ≥1 wk before travel) (reduced efficacy)	2nd dose ≥ 1 wk before travel, counsel on reduced efficacy
Rabies	4 doses (0, 7, 21, 28 days) + serology	4 doses (0, 3, 7, 14 days) + serology	Neutralizing titer ≥ 0.5 IU/mL, last dose ≥ 1 wk before travel
MMR/Varicella	Contraindicated (severe)/individualized (HIV, asplenia)	Contraindicated (severe)/individualized (HIV, asplenia)	Specialist clearance, IgG titer if indicated
Zoster (RZV)	2 doses (0, 2–6 mo)	Not accelerated	2nd dose ≥ 2 wks before travel, counsel on reduced efficacy

**Table 6:** Warning signs of illness.

Fever	Even Low-Grade
Diarrhea	especially persistent, bloody, or associated with dehydration
Respiratory symptoms	cough, shortness of breath, chest pain
Other acute changes	skin lesions, confusion, jaundice, or focal pain

## Data Availability

No new data were created or analyzed in this study. Data sharing is not applicable to this article.

## Abbreviations

The following abbreviations are used in this manuscript:

HSCT	Hematopoietic Stem Cell Transplant
GvHD	Graft-versus-Host Disease
HIV	Human Immunodeficiency Virus
CMV	Cytomegalovirus
HSV	Herpes Simplex Virus
VZV	Varicella-Zoster Virus
EBV	Epstein–Barr Virus
ART	Antiretroviral Therapy
CDC	Centers for Disease Control and Prevention
ACIP	Advisory Committee on Immunization Practices
IDSA	Infectious Diseases Society of America
RIV	Recombinant Influenza vaccine
LAIV	Live Attenuated Influenza vaccine
PCV20	20-valent Pneumococcal Conjugate vaccine
PCV15	15-valent Pneumococcal Conjugate vaccine
PPSV23	23-valent Pneumococcal Polysaccharide vaccine
RZV	Recombinant Zoster vaccine
MMR	Measles, Mumps, Rubella vaccine
ViCPS	Vi Capsular Polysaccharide vaccine
IPV	Inactivated Polio vaccine
YEL-AVD	Yellow Fever Vaccine-Associated Viscerotropic Disease
YEL-AND	Yellow Fever Vaccine-Associated Neurologic Disease
MenACWY	Quadrivalent Meningococcal Conjugate Vaccine
MenB	Serogroup B Meningococcal Vaccine
IG	Immunoglobulin
IgG	Immunoglobulin G
PrEP	Pre-Exposure Prophylaxis
PEP	Post-Exposure Prophylaxis
STI	Sexually Transmitted Infection
STD	Sexually Transmitted Disease
HPV	Human Papillomavirus
HAV	Hepatitis A Virus
HBV	Hepatitis B Virus
HCV	Hepatitis C Virus
TB	Tuberculosis
PCR	Polymerase Chain Reaction
IGRA	Interferon-Gamma Release Assay
NAAT	Nucleic Acid Amplification Test
IIV	Inactivated Influenza vaccine
IM	Intramuscular
